# Conservation of terrestrial invertebrates: a review of IUCN and regional Red Lists for Myriapoda

**DOI:** 10.3897/zookeys.930.48943

**Published:** 2020-04-28

**Authors:** Manoela Karam-Gemael, Peter Decker, Pavel Stoev, Marinez I. Marques, Amazonas Chagas Jr

**Affiliations:** 1 Programa de Pós Graduação em Ecologia e Conservação da Biodiversidade, Universidade Federal de Mato Grosso, Cuiabá, Mato Grosso, Brazil; 2 Senckenberg Museum of Natural History Görlitz, Am Museum 1, 02826 Görlitz, Germany; 3 National Museum of Natural History, Sofia, Bulgaria; 4 Pensoft Publishers, Sofia, Bulgaria; 5 Programa de Pós Graduação em Zoologia, Universidade Federal de Mato Grosso, Cuiabá, Mato Grosso, Brazil; 6 Laboratório de Taxonomia e Sistemática de Artrópodes Terrestres, Departamento de Biologia e Zoologia, Universidade Federal de Mato Grosso, Cuiabá, Mato Grosso, Brazil

**Keywords:** Arthropoda, Chilopoda, Diplopoda, extinction, national red lists, Pauropoda, risk assessment, Symphyla, threatened species

## Abstract

Red Listing of Threatened species is recognized as the most objective approach for evaluating extinction risk of living organisms which can be applied at global or national scales. Invertebrates account for nearly 97% of all animals on the planet but are insufficiently represented in the IUCN Red Lists at both scales. To analyze the occurrence of species present in regional Red Lists, accounts of 48 different countries and regions all over the world were consulted and all data about myriapods (Myriapoda) ever assessed in Red Lists at any level assembled. Myriapod species assessments were found in eleven regional Red Lists; however, no overlap between the species included in the global IUCN Red List and the regional ones was established. This means that myriapod species considered threatened at regional level may not be eligible for international funding specific for protection of native threatened species (more than US$ 25 million were available in the last decade) as most financial instruments tend to support only threatened species included in the IUCN Red List. As the lack of financial resources may limit protection for species in risk of extinction, it is urgent to increase the possibilities of getting financial support for implementation of measures for their protection. A Red List of all Myriapoda species recorded in Red Lists at national or local (596) and global (210) scales totaling 806 species is presented. This list shows for the first time an overview of the current conservation status of Myriapoda species. Here, the urgent need of establishing a Myriapoda Specialist Group in the Species Survival Commission of IUCN is also stressed.

## Introduction

Biodiversity conservation is an applied science which involves several tools and approaches to avoid species extinction and protect environment as a whole. The approaches for conservation planning may vary in scale and extent (Pressey and Bottrill 2009), but they need to rely on rigorous evidence on species and ecosystems involved (Cook et al. 2010). Specifically, evidence-based wildlife management requires reliable information on the conservation status and the extinction risk of species (Charra and Sarasa 2018).

The most widely recognized assessment of the conservation status of species is the Red List of Threatened Species, established by the International Union for Conservation of Nature (IUCN) in 1964 (Charra and Sarasa 2018). Since then, IUCN has been developing and updating a global list of threatened species and a methodology for species assessments. The organization has also been investing efforts in expanding its taxonomic expertise. The IUCN Red List of Threatened Species plays an important role of a global database of the conservation status of various organism groups (IUCN 2019a).

In the past decades, several countries have elaborated own lists of threatened species, often based on IUCN guidelines for species assessments (IUCN 2012; IUCN 2019b). The national and regional lists are essential for the implementation of local conservation actions, where policy is often implemented (IUCN 2019a). They also supply national governing bodies with information of both scientific and political relevance regarding the state of biodiversity, and as such could be a valuable resource for conservation planning (Zamin et al. 2010). Red Lists that focus on specific areas are of particular importance in aiding national reporting to international conventions with specific biodiversity targets, such as the Convention on Biological Diversity and the Sustainable Development Goals (IUCN 2019a). Furthermore, the information from local species assessments is important for developing species and ecosystem-based strategies for climate change adaptation (Zamin et al. 2010).

To meet the needs of the scholars, some scientific journals have developed specific publication types compliant with the IUCN Red List species assessments (Cardoso et al. 2016). Among the invertebrate experts, arachnological community is especially active in publishing species assessments at various scales in the form of academic papers (Seppälä et al. 2018, Branco et al. 2019, Fukushima et al. 2019). Similar surveys have recently been published for moths, cave-dwelling arthropods (Borges et al. 2018, 2019) and an endangered species of rattan palm from Africa (Cosiaux et al. 2017) to name a few. Specific software has also been developed to ease the species assessments (Cardoso 2017).

**Figure 1.**
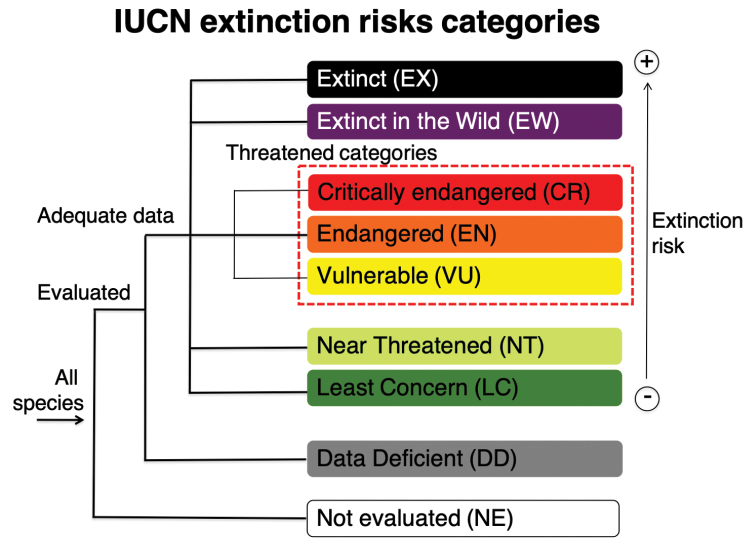
IUCN extinction risks categories for global assessments. Threatened categories are within dashed red line. (Modified from IUCN 2019).

The IUCN global species conservation assessments are based on objective criteria which classify taxa into nine clearly defined categories (Fig. 1). Of them, three categories concern species with higher extinction risk: Critically Endangered (CR), Endangered (EN), and Vulnerable (VU). The other categories refer to Extinct (EX) or Extinct in the Wild (EW) species; species that are close to become threatened (Near Threatened – NT); species that do not qualify for threatened nor NT categories (Least Concern – LC); species without sufficient data available for assessment (Data Deficient – DD); and Not Evaluated species (NE). At smaller scale, two other categories are being introduced: Regionally Extinct (RE), for species extinct in the wild within the respective region, and Not Applicable (NA), for species that do not qualify for assessment at a regional level (i.e., introduced taxa).

Until 2019, IUCN Red List had already assessed 71% of vertebrate species, 11% of plant species, and 2% of invertebrate species in the world (IUCN 2019a). In latest IUCN report (2019a) 49% of the species considered as threatened are animals and 51% are plants (fungi account for less than 1%). Among threatened fauna, 63% are vertebrates and 37% are invertebrates. The species groups covered by the IUCN Red List are biased towards terrestrial and in particular forest ecosystems (IUCN 2019a). Estimated to represent around 97% of all animals on the planet, invertebrates are the least conservational explored group and currently form only 31% of all animal assessments in the Red List (IUCN 2019a). There is a taxonomic bias in IUCN Red List that excludes species with small body sizes, narrow distribution ranges and low dispersal abilities, which constitute the vast majority of the planet’s biota, particularly local endemics (Cardoso et al. 2011). At national level, invertebrates are also among the least represented as regards the taxonomic coverage (Zamin et al. 2010). The low number of experts working with the group and, consequently, the lack of data on historic population and distribution trends may help to explain the taxonomic bias towards the invertebrates in Red Lists.

Myriapoda is a group of terrestrial arthropods of high ecological importance. It comprises four, well defined classes: Diplopoda (millipedes), Chilopoda (centipedes), Symphyla (symphylans), and Pauropoda (pauropodans). As top invertebrate predators, centipedes drive ecosystem function, for example, by regulating decomposer populations (Phillips et al. 2019). Millipedes are important ecosystems decomposers, as their impact on fragmenting leaf litter increases surface area for microbial processing and, consequently, soil development and mineralization of nutrients for plant growth (Snyder and Hendrix 2008). The other two classes are less diverse and while symphylans could be pests, especially by eating seeds and roots of young crops, pauropodans are of little agricultural importance. In contrast to many other Arthropoda, myriapods show a very low tendency to disperse, which results in high species endemism (Voigtländer et al. 2011). The current version of IUCN Red List (2019a) comprises 210 myriapods assessments (200 millipede species and 10 centipede species), which represents only 1% of all described species (circa 17 000 species; Sierwald and Spelda 2019). Among assessed species, 44% are listed in categories of higher threat and 16% are considered Data Deficient (IUCN 2019a). However, the actual number of threatened species for any group is often uncertain because it is not known whether Data Deficient species are actually threatened or not.

Several countries have assessed and listed myriapod species in their own national or regional census (Gärdenfors 2010; MMA 2014; Henriksen and Hilmo 2015). Although regional lists are essential for presenting evidences for local conservation decisions and therefore must be presented primarily in local language, they are often published in obscure outlets or exclusively in local languages, which makes their finding and comprehension difficult for a meta-analysis. In general, when pertinent information on biological diversity is too sparse or scattered it may not to be of practical use for a global scale analysis. In such cases the information needs to be gathered through systematic efforts to strengthen the entire research process (National Research Council 1992). The IUCN Red List accepts only global level assessments, but regional evaluations can also be included if they refer to single-country endemics for instance and the applied assessment process follows strictly the IUCN guidelines. As a whole, local red lists are not taken into consideration by the global database of IUCN. For instance, myriapod endemic species listed in the Brazilian Red List are not included in IUCN global database (Karam-Gemael et al. 2018a). Since Brazil’s list was elaborated under the IUCN guidelines, why the endemic species assessed are not included in the IUCN Red List? There is no mechanism that IUCN SSC evaluate already existing national or regional Red Lists or at least this seem not to be in the agendas of the expert groups.

The addition of endemic species assessed regionally in the IUCN Red List has two major applications and benefits. On one hand, it would expand the taxonomic coverage of a given organism group, thus having direct implications for the species management both at local and global levels. On the other hand, it also allows endemic threatened species to be eligible for international funding (Karam-Gemael et al. 2018a). There are several specific funding schemas for conservation of threatened species listed by IUCN. Increasing the possibility of getting financial support for applied research and conservation measures is especially important as naturally this is an integral limitation for species in risk of extinction (Zamin et al. 2010). This may be particularly true for terrestrial invertebrates which are usually of lower conservation priority. The invertebrate protection usually concerns the conservation of habitats, and thus the whole community occurring in a given habitat could benefit the measures. Attracting funds for “umbrella species” which some invertebrate species could be, would mean to invest in the protection of the habitat as a whole for the benefit of other threatened taxa. In Brazil alone, the occurrence of the threatened endemic velvet worm *Epiperipatus acacioi* (Marcus & Marcus, 1955) in Minas Gerais (Brazil) justified the creation of the first Ecological Station in the state (ESEC Tripuí), a very restrictive category of federal protected area according to the Brazilian legislation (FEAM 1995). Another similar case in Brazil is a protected area created for dragonflies (RVS Libélulas da Serra de São José; Diário do Executivo 2004). Having a mechanism for getting species assessed at regional/national/local scale on the IUCN Red List would boost such surveys.

The main aim of the present study is to analyze the congruence between the invertebrate species assessed in regional Red Lists against the IUCN Red List, using Myriapoda as a case study.

## Materials and methods

We have assembled a global list of Myriapoda species in Red Lists irrespective of their scale. For data collection, we conducted a global survey for national/regional lists between May and July 2019. First, we searched for Myriapoda species in the Red Lists available at the National Red List website database (Zoological Society of London 2019). We also used the snowball sampling method to obtain a sample of eligible countries from different continents. This method helps to increase the diversity of sampling (Rossi et al. 2004), but its limitations prevent the estimation of how representative a sample is (Walsh et al. 2015). Once we found an eligible Regional Red List, we looked for Myriapoda species assessed. From each list we collected the species name, extinction risk category, language of publication, and the methodology used for assessment (based on IUCN guidelines or not). To avoid missing regional Red Lists with Myriapoda species listed due to the limitation of snowball sampling, we carried out an online survey asking the experts associated to the International Society for Myriapodology if their country/region had ever assessed myriapod species in their Red Lists. Finally, for IUCN Red List data we performed an advanced search at its website and then downloaded all myriapods assessments in an Excel spreadsheet with all species assessed and its extinction risk categories (version 2019-2).

For data analysis, we joined data from all regional Red Lists where myriapod species were found and the results from the IUCN Red List search to create a unique database with all the species names of Myriapoda assessed, the current valid taxon name, its extinction risk categories according to IUCN, the original category (if differing to IUCN), methodology used and the (literature) source of the data. We then analyzed: a) which species from the IUCN Red List were also present in regional Red Lists (congruence among the lists); b) which species are represented in more than one Regional Red List; c) the proportion of each extinction risk category; d) correlations between: growth in GDP (World Bank 2019), protected areas network coverage (World Bank 2019), deforestation rate (Mongabay 2019), CO2 emission rates (World Bank 2019) for each country included as well as the proportion of threatened species recorded for that country; e) which species are endemic; we considered endemic a species occurring only in the country where it is listed. Taxonomic and geographic data for most species were gathered from the taxonomic online catalogues Millibase (Sierwald and Spelda 2019) and Chilobase (Bonato et al. 2016).

The German Red List uses some additional refined categories which have no analogue in the IUCN Red List. The categories “extreme rare species with geographic restriction” (R) and “threat of unknown extant” (G), which are situated between the IUCN “near threatened” (NT) and “vulnerable” (VU) are translated according to Ludwig et al. (2009) to NT for the former and VU for the latter. In the Slovenian Red List, the categories “rare species” (R) and “undetermined species” (I) were translated with IUCNNT, and the category “unknown species” (K) with data deficient (DD).

## Results

**Figure 2.**
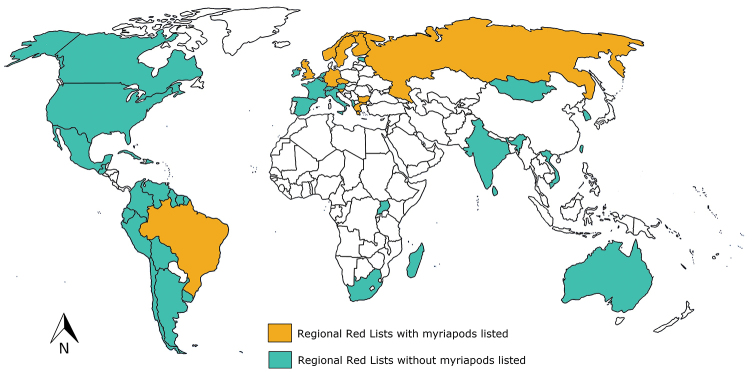
Countries included in this study. In orange, countries consulted that have their own local lists and myriapods are listed: Brazil, Bulgaria, Czech Republic, Finland, Germany, Greece, Norway, Slovenia, Russia, Sweden, UK. In green, countries consulted that have their own local lists, but there are not myriapods listed: Albania, Argentina, Australia, Austria, Belgium, Bolivia, Canada, Chile, Colombia, Cuba, Dominican Republic, Ecuador, Estonia, France, French Guiana, Guatemala, Guiana, India, Italy, Ireland, Mexico, Madagascar, Mongolia, Netherlands, Peru, Republic of Korea, South Africa, Spain, Sri Lanka, Suriname, Switzerland, Taiwan, Uganda, Uruguay, USA, Venezuela, Vietnam.

We consulted regional Red Lists of 48 countries from: South America (12), Europe (19), Africa (3), Asia (7), North America (3), Central America (3), and Oceania (1) (Fig. 2). We found Myriapoda species assessments in 23% of them (Table 1).

**Table 1.** National/regional Red Lists with Myriapoda species assessments included in this study. Documents are presented in order of publication year. Complete literature information of each one is given in Suppl. material 1.

Country	Region	Year	References
Brazil		2003, 2014	MMA (2003), MMA (2014)
Bulgaria		2011	Golemansky (2011)
Czech Rep.		2005, 2017	Kocourek (2005), Hejda et al. (2017)
Finland		2019	Hyvärinen et al. (2019)
Germany		2016	Decker et al. (2016), Reip et al. (2016)
	Baden-Württemberg	1998	Spelda (1998)
	Bavaria	2004	Spelda (2004)
	Saxony-Anhalt	2004; in press	Voigtländer (2004a), Voigtländer (2004b), Lindner et al. (in press), Voigtländer et al. (in press)
Greece		2009	Legakis and Maragos (2009)
Norway		2006, 2010, 2015	Djursvoll and Meidell (2006), Dursvoll (2010), Henriksen and Hilmo (2015)
Russia	Altai	2016	Altai University (2016)
	Republic of Komi	2019	The Red Data Book of the Rep. of Komi (2019)
	Sevastopol	2019	The Red Data Book of Sevastopol (2019)
	Tver Area	2016	The Red Data Book of the Tver Area (2016)
Slovenia		1992, 2002	Kos (1992), Mrsic (1992), Official Gazette of RS No. 82/2002 (2002a/b)
Sweden		2010	Gärdenfors (2010)
Great Britain		2015	Natural England (2015)

The survey resulted in assembling a spreadsheet comprising 596 taxa assessed in regional lists. On the other hand, the IUCN Red List of Threatened Species records 210 species of Myriapoda. There are no species in common between the local lists and the global one, therefore, there is no congruence among the lists analyzed at species level (Table 2).

**Table 2.** Diversity of Myriapoda in the worldwide Red List of threatened species presented here. Includes data from IUCN Red List (2019) and from Regional Red Lists from eleven countries compiled for this study (for countries included see Fig. 2).

Class	Taxonomic level	IUCN Red List	Regional Red Lists	Shared taxa
** Diplopoda **	Families	12	50	**4**
	Genera	35	207	0
	Species	200	654	0
** Chilopoda **	Families	5	13	**4**
	Genera	5	32	0
	Species	10	130	0
** Pauropoda **	Families	0	1	0
	Genera	0	6	0
	Species	0	14	0
** Symphyla **	Families	0	2	0
	Genera	0	4	0
	Species	0	8	0

A global list with all myriapod assessments is presented in Suppl. material 1. It considers both the species present in regional Red Lists and in the IUCN Global Red List, and totals 806 species belonging to the four Myriapoda classes: Diplopoda (81%), Chilopoda (16%), Pauropoda (2%), and Symphyla (1%). Among the Diplopoda, species from 13 orders (out of 16 worldwide) have been assessed, of which Polydesmida (27%) and Spirobolida (16%) are the most assessed orders. Among Chilopoda, species from four (out of five) orders have been assessed: Lithobiomorpha (46%), Geophilomorpha (41%), Scolopendromorpha (11%), and Scutigeromorpha (2%). Only the order Craterostigmomorpha, which is restricted to Tasmania and New Zealand, was not assessed.

Although the number of species assessed is 806, the total of assessments found is 1289, due to: a) species assessed in two, three or four countries; b) species reassessed in the same country; and c) species listed in NE (Not Evaluated) and NA (Not Applicable) categories in Germany’s and Norway´s list (even not being assessments itself, species listed in these categories are included in the list).

**Figure 3.**
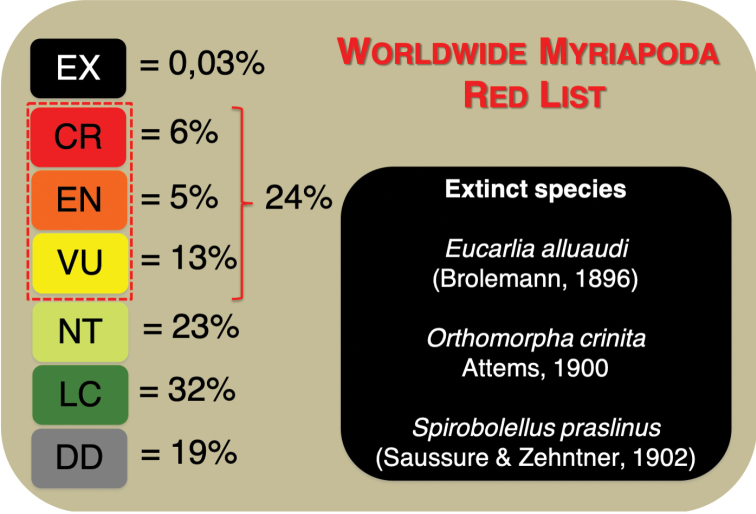
Proportion of extinction risk categories of the Worldwide Myriapoda Red List presented here. Includes data from regional Red Lists from eleven countries and from IUCN Red List of Threatened Species. Threatened categories are within dashed red lines.

Among all myriapods assessed (local lists and IUCN Red List), 24% are considered as threatened (Critically Endangered (CR), Endangered (EN) or Vulnerable (VU)). According to the assessments three species are considered extinct. Species assessed as Data Deficient account for 19% (Fig. 3). A significant number of myriapods classified as Least Concern (LC) (32%; Fig. 3) comes from the regional Red Lists analyzed (91%), rather than from the IUCN Red List (9%). Brazil and Germany are responsible for 80% of all species assessed as LC. Among the species assessed only by regional lists, 36% are in threatened categories (CR, EN, VU; Table 3). Brazil and Germany are the countries with more myriapods assessed.

**Table 3.** Total of Myriapoda assessments per country per extinction risk category. The column Total threatened species sums up the CR, EN, and VU categories. Table is sorted according to Total Threatened Species in decreasing order. Brazil’s assessments include data from the 2014 Red List plus the troglobitic species assessed in 2018 but not published yet.

Country	EX	CR	EN	VU	NT	LC	DD	NA	NE	Total threatened species	Total assessments
Slovenia		4		86	117		17			90	224
Germany		4	13	11	85	207	51		3	28	374
Czech Republic		5	2	25	15		9			32	56
Brazil		11	9	12	1	119	98			32	250
Russia		2	1	8						11	11
Bulgaria		4								4	4
Greece		3	1							4	4
UK			2	2	6		9			4	19
Sweden		1		2	6		6			3	15
Norway			1	6	18	49	17	11	10	7	112
Finland		1			3		6			1	10
**IUCN**	**3**	**37**	**37**	**19**	**42**	**35**	**37**			**93**	**210**
Total	3	72	66	171	293	410	250	11	13	309	1289

The proportion of threatened myriapod species in the regional Red Lists analyzed was significantly and positively correlated with the growth in GDP from the countries included (r-value = 0.71) (those countries which have assessed Myriapoda in their national Red Lists; see the list in Fig. 2). Correlations between threatened species and: protected areas network coverage (r-value = 0.84), deforestation rate (r-value = 0.02), and CO2 emissions (r-value = 0.05) were also all positive.

Among the eleven countries, eight applied the IUCN methodology for the assessments. Russia and Slovenia applied their own methodology, but former used methodology corresponding to the categories of IUCN. Slovenia’s methodology is also based on equivalent categories. The methodology used by Germany is a modified IUCN version. Among the Red Lists of eleven countries with myriapods assessed, 54% were published only in the native language of the country, with no English version or abstract available.

## Discussion

The analysis of similarity of regional Red Lists and the IUCN Red List for myriapods showed that endemic species already assessed at national/local level have not been included in IUCN global database. Also, there is no congruence on species level between regional lists and the IUCN Red List for myriapods. The data presented here increase the awareness about the dissimilarity between regional Red Lists and the IUCN Red List, as it corroborates the results found for Brazilian myriapods (Karam-Gemael et al. 2018a).

Two main points arise from the incongruity between regional Red Lists and IUCN Red List found here. Firstly, Myriapoda seem to be largely neglected by the terrestrial invertebrate censuses of IUCN, which may be true also for other arthropod groups. Another reason probably is that only very few specialists working on Myriapoda worldwide. Then, the few species specialists existing have the responsibility to work on regional and/or global assessments, even if they only assess members of their scope taxa. Besides that, it is also relevant to consider that the scientific evidences presented in regional lists are scattered in several documents with different formats and languages worldwide. Of course, regional Red Lists should be presented primarily to the national politics, decision makers and NGOs without any language burdens and in official governmental publication. However, in Germany, for example, the nationwide Red List experts have put pressure on the responsible federal office so that an English abstract will be available in the next version of the German Red Lists and the publication will now also be freely available as a PDF. Easy access and English language will help to integrate data resources and to abroad IUCN Red List coverage. As IUCN list works as an objective indicator of the health of the world’s biodiversity showing which wild species are threatened, one of its urgent targets is to fund the assessment of taxa not yet well represented on The IUCN Red List (IUCN 2019a). The worldwide list presented here can help to partially fill in IUCN Red List gap in invertebrate’s coverage, as current version of IUCN Red List presents only 1% of known myriapods, and the total number of worldwide assessed species (806) presented here represents 5% of all species known for the group. This will help expand coverage to a more representative subset of the planet’s biodiversity.

In fact, it is expected that taxonomic groups not represented among the IUCN Specialists Groups will receive less attention and consequently will be less represented in the IUCN Red List. Currently there are 13 terrestrial invertebrates Specialists Groups, besides Cave Invertebrate, South Asian Invertebrate, Terrestrial and Freshwater Invertebrate, and Mid Atlantic Islands Invertebrate groups (IUCN 2019a). When submitted for evaluation to IUCN, myriapod species assessments would be handled by one of the groups focused on invertebrate mentioned above. Otherwise, the assessments are forwarded to the Invertebrate Sub-Committee. The Species Survival Commission Specialist Groups are considered the Red List Authorities (RLA) for the species in their remit within IUCN (IUCN 2019a). Among the responsibilities of RLA specific for taxonomic groups are establishing mechanisms for assessing and regularly reassessing species, undertaking assessments at the global level. Considering the diversity of terrestrial invertebrates and their poor representation in the IUCN Red List, it is strongly recommended the designation of more invertebrate specialist groups, and in particular Myriapoda Specialist Group.

The second main point that arises from the incongruity between the lists is that endemic myriapod species assessed as threatened in regional Red Lists that followed IUCN guidelines are currently not eligible for international funding. Most international funding organizations support conservation activities only directed to IUCN listed threatened species. Among 596 Myriapoda species compiled from regional Red Lists, 36% are assessed in threatened categories (CR, EN, and VU). It means that these species could be eligible for international funding if they were also listed by IUCN. In the last decade, international biodiversity conservation organizations directed more than US$ 25 million specifically to fund projects with species assessed as threatened by IUCN Red List (Karam-Gemael et al. 2018a). Considering the significant taxonomic gap for invertebrates in IUCN Red List, it is likely that a large amount of species of other invertebrate groups may also be find in threatened categories in regional lists but are missing in the IUCN Red List. Then, the scenario discussed here may be helpful when discussing terrestrial invertebrate’s conservation in general.

Brazil and Germany are together responsible for 80% of all species currently assessed as Least Concern (LC) in the worldwide list of Myriapoda presented here. The category is applied to taxa that do not qualify as Threatened or Near Threatened. However, it is important to emphasize that “least concern” simply means that, in terms of extinction risk, these species are of lesser concern than species in other threat categories. It does not imply that these species are of no conservation concern (IUCN 2019b). According to IUCN criteria, species known only from the type locality may be classified as LC if the area is relatively well known and there are no plausible threats. However, it also could be a product of insufficient data about the species or the area where it occurs. Then, similarly to Data Deficient species (which may hide threatened species in function of no information about them), species assessed as LC may be considered as threatened in a near future when more information about the group or the area is brought up.

More than half of the lists where we found myriapods assessments were published in the native language of the country only, with no English version. This is an important barrier to biodiversity conservation and science in general, which has English as its universal language. We have also found several different formats of publishing the data. Although some countries (Russia, Slovenia, and Germany) apply similar categorization, no IUCN standardized protocols for assessments were used. It means that data from these countries’ red lists may be informative and sufficient for inform local management needs, but cannot be linked to or incorporated in the IUCN Red List. Therefore, using different methodologies to assess species extinction risks does not allow comparability and no sharing of information between lists, because including species assessed locally in IUCN Red List demands that assessments followed IUCN guidelines strictly. IUCN categories and criteria have a history of versions throughout the decades, as it has been revised under extensive consultation processes and workshops in several occasions (IUCN/UNEP et al. 1987, Mace and Lande 1991, IUCN 2001, IUCN 2003, IUCN 2019b). Hence, it is important to keep in mind that even IUCN Red Lists are not really 100% comparable and regional Red Lists according to the IUCN need to be carefully checked about which version and if modified differences need to be mentioned. And even when assessments follow the same methodology, the publication format may vary in: the associated data included; the arrangement of presenting species (by taxonomic group or by assessed category); the format itself (a downloadable PDF, listed in a webpage or not available online). Altogether, these publications variations and the native language were very time consuming to overcome when gathering data. When science data is freely and easily available (in English, gathered and standardized, with online open access) it creates new opportunities for integrating data between research projects and analyzing data in additional ways. The long-term availability of data is especially important in conservation science because field data can be costly to collect. In addition, historic data, especially on threatened species and their associated biota, become more valuable over time (Costello and Wieczorek 2014).

Although it may seem illogical, the countries with higher number of threatened myriapods are also those with bigger proportion of protected areas (PAs). This is also supported by the notion that PAs are not designed to really make a difference in conservation (Pressey et al. 2015). The reasons for this discrepancy could be that those countries are more sensitized about habitat loss and spend more money on biodiversity surveys and/or have better research infrastructure to assess endangerment of habitats and taxa. Likewise, in Brazil alone, current PAs network fails to protect the majority of endemic species as demonstrated by vertebrates, arthropods, and angiosperms (Oliveira et al. 2017). On the other hand, it was expected that correlations among myriapods threatened species and deforestation and CO_2_ emissions rates would be positive, as deforestation accurately predicts threat to endemic species (Brooks et al. 1999). Similarly, GDP growth is also higher where there are the majority of threatened species. In fact, it is expected that emerging economies present a high number of threatened species, as they are in general in tropical areas with high endemism levels and facing the pressure of human population growth, intense urbanization processes and increase area of intensive land use.

### Connecting regional Red Lists

Analyzing several regional Red Lists showed that it may turn out being isolated from other local lists, even when considering neighboring countries. In continents like Europe, for example, Red Lists of countries with low (or no) endemism may assess the same population of a given species considering data and threats specific for its area, but missing information that impacts the same population from its neighboring countries.

Our results show that some species were classified in different categories in neighboring countries. For example, the millipede *Leptoiulus cibdellus* (Chamberlin, 1921) is assessed as Least Concern (LC) in Germany (Reip et al. 2016), as Endangered (EN) in the federal state Saxony-Anhalt in Germany (Voigtländer et al. in press), and Vulnerable (VU) in the Czech Republic (Hejda et al. 2017). Considering that Germany and Czech Republic share a border, it is possible that both countries assessed the same population simultaneously. Therefore, local conservation management may need to broaden the targets of actions and programs to a regional level, as interactions among sub-units should be carefully considered when planning conservation actions (IUCN 2019b). It is also interesting to note the different threats operating in each country, as the threats also impact conservation status assessments. Although the IUCN methodology and criteria may be applied at any geographical scale (considering the guidelines for regional assessments), application within very restricted geographical areas is strongly discouraged by IUCN (IUCN 2012). In a small region, a wide-ranging taxon will frequently exchange individuals with neighboring regions, leading to unreliable assessments (IUCN 2012). Then, expanding the geographic range of the assessment brings a broader overview of the group and may be a stronger evidence to inform conservation decision, even in local, regional or global scales. It means that local assessments should always be interpreted alongside other available assessments in the region/continent.

It is necessary and important to reassess species every five to ten years, as risk of extinction may change due to increase of data (e.g., distribution, ecology), change in land use or habitat size. Such case can be illustrated in the case of *Thalassisobates littoralis* (Silvestri, 1903) on the Red List of Norway. In 2006 it was assessed as Vulnerable (VU), 2010 as Near Threatened (NT) and 2015 as Endangered (EN) (Djursvoll 2006, Djursvoll and Meidell 2010, Henriksen and Hilmo 2015).

### Connecting regional and global Red Lists

Both local and global lists are essential for informing evidence-based conservation management. Joined, they can lead to new possibilities to work with threatened species, including increasing financial resources. But who is responsible for connecting local and global lists?

Scientists working with endemic species could take their part in this task, as they are often those who participate of species assessments in regional/national level. Scientists are (or should be) aware that including species in Red Lists may help to approximate evidence-based science to practice, once Red Lists assessments are based on evidences and also that threatened species may inform the process of priority setting for conservation management (see next section). Research groups working with endemic species (usually coordinated by scientists) would be directly impacted with the potential raise in projects funding. Then, we suggest two ways that scientists could contribute with the connection between local and global lists. First, scientists could collectively demand from the environmental agency in charge of the national lists workshops in a given country that the final assessments files are translated into English for publication in the IUCN Red List. In general, the communication between academia and environmental managers is poor and conservation decisions often lack scientific evidences (Karam-Gemael et al. 2018b). Then, this would be an opportunity of narrowing the flow of demands and information between science and practice. Second, scientists could help to improve taxonomic gaps in IUCN List by including the assessment of the species they are working with in their research projects. When it is not a large amount of species, the assessment process and its preparation in the standardized IUCN assessment file may not be a huge task. Also, scientists working with ecology and biodiversity conservation usually are familiar with the literature, the language and the methods applied in the assessment process.

IUCN welcomes Red Lists assessments of endemic species resulting from projects carried out by academia and from national Red List initiatives. The process for submitting data is formalized and follows several steps of revisions, data validation and final checks by IUCN staff before publication in the IUCN Red List. The “Guidelines for application of IUCN Red List criteria at regional and national levels” is available online in several different languages. It allows for experts working with endemic species include the assessment of its targeted species in their projects. Adding more species assessments in the IUCN Red List would result in increasing: 1) taxonomic coverage of the group knowledge; 2) coverage of the global list; 3) funding opportunities; 4) scientific evidences for conservation science.

Besides scientists, the governments and institutions supporting national Red Lists initiatives could also take their part on helping to connect local and global lists. When planning national assessments workshops, they could include in the project budget the translation of the files and the supporting information to English.

It is important to notice that the connection between Red Lists (among Regional lists and between Regional and IUCN Red List) based on IUCN methodology depends on using the same updated methodology and standards when analyzing and publishing data. It helps to ensure that local lists are comparable and promotes the sharing of species information between neighboring countries. Using a comparable methodology also allows an easier flow of information between the regional and global levels. A regional approach to identifying threatened species complements the global Red List and provides information at an appropriate scale for international conservation treaties (for example, the Bern Convention) and legislation (e.g., the EU Habitats Directive) that have a regional focus (IUCN 2019a).

### Priority for conservation action

Although the species listed in threatened categories may be those for which the risk of extinction is higher, it is essential to point out that assessment of extinction risk and the process of setting conservation priorities are related but different processes. The extinction risk assessment shows the likelihood of extinction of the taxon, and as such it is part of the process of setting conservation priorities, but alone it is not sufficient to determine conservation priorities. Setting conservation priorities should also takes into account other factors rather than the extinction risk, such as ecological traits, economic and cultural values, the probability of success of conservation actions, availability of funds and specialists to carry out the actions, legal context for conservation of threatened species (IUCN 2012).

Especially when considering regional/national/local assessments for non-endemic species, priorities setting should also take into account not only conditions within the region but also the status and population size of the taxon at the global level. Then, IUCN recommends that the publication of regional assessments of non-endemic taxa should include the global assessment and the proportion of the global population occurring within the region (IUCN 2012).

## Supplementary materials

Supplementary material 1 Worlwide Myriapoda assessments Data type species data https://binary.pensoft.net/file/404970This dataset is made available under the Open Database License (http://opendatacommons.org/licenses/odbl/1.0/). The Open Database License (ODbL) is a license agreement intended to allow users to freely share, modify, and use this Dataset while maintaining this same freedom for others, provided that the original source and author(s) are credited.Manoela Karam-Gemael, Peter Decker, Pavel Stoev, Marinez I. Marques, Amazonas Chagas Jr
